# Right Pulmonary Artery Agenesis in an Adult: A Case Report of Silent Pulmonary Hypoplasia

**DOI:** 10.7759/cureus.88615

**Published:** 2025-07-23

**Authors:** Mohamed Ijim, Oussama Fikri, Lamyae Amro

**Affiliations:** 1 Pulmonology Department, University Hospital Center Mohammed VI, Arrazi Hospital, Faculty of Medicine and Pharmacy of Marrakech, Morpho Sciences Research Laboratory, Cadi Ayyad University, Marrakech, MAR

**Keywords:** agenesis, artery, congenital, hemoptysis, hypoplasia, lung

## Abstract

Pulmonary artery agenesis is a rare congenital malformation and most often affects the right pulmonary artery. It is associated with other congenital anomalies such as tetralogy of Fallot, or it can be isolated and revealed in a less dramatic presentation in adulthood. We report in this work a case in an adult. This is a 40-year-old patient admitted for chest tightness and pain. The clinical examination was normal. Chest CT angiography and echocardiography confirmed the diagnosis of right pulmonary artery agenesis without other associated malformations. The patient has been followed for one year with good progress. Early diagnosis allows for planned and appropriate management.

## Introduction

Unilateral pulmonary artery agenesis (UAPA) is a rare congenital malformation, and the number of published cases varies between studies. A 2011 systematic review identified 352 cases of UAPA, including 237 associated with other congenital cardiac anomalies (such as tetralogy of Fallot or ventricular septal defects) [1-3]. This study remains the most comprehensive reference we found. Isolated UAPA (without associated cardiac anomalies) is estimated to affect one in 200,000 to 300,000 adults, according to epidemiological data [2,3]. UAPA is more common on the right (60% of isolated cases), while left-sided involvement is often associated with other cardiovascular malformations (80% of cases) [2,3]. There are no specific symptoms for this malformation. Prognosis depends on the extent of hypoplasia and the presence of other congenital abnormalities.

Although more than 350 cases have been documented as of 2011, there are no standardized recommendations for the specific management of UAPA.

## Case presentation

A 40-year-old patient with no particular medical history presented with progressive dyspnea on exertion, associated with mild, atypical, intermittent chest pain that had been evolving for four weeks. He had no toxic habits or comorbidities and maintained intermittent physical activity of moderate intensity. The patient had not reported a cough. Dyspnea was grade I according to the modified Medical Research Council (mMRC) classification, associated with chest pain that was mild in intensity, intermittent, and not clearly triggered by any specific factors. There was no history of fever, wheezing, anorexia, or weight loss.

**Figure 1:**
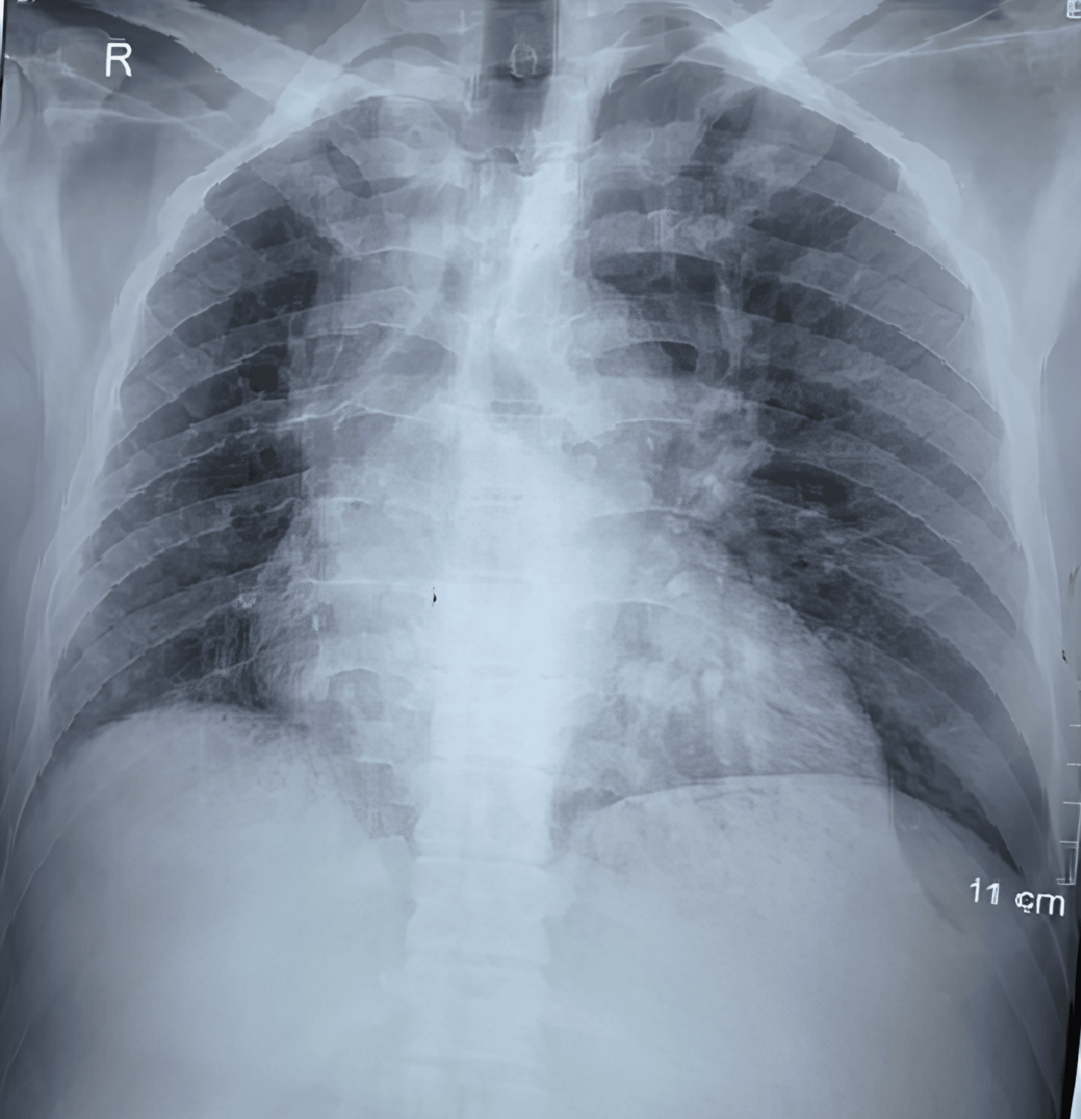
Front chest X-ray: retraction of the left pulmonary field, reduction of the left hilum, and hyperinflation of the right side

**Figure 2:**
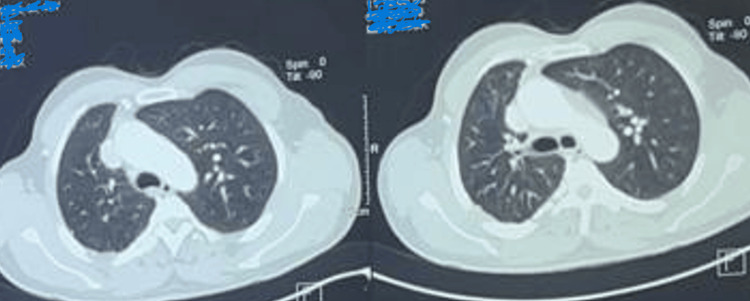
Chest CT parenchymal section: right lung hypoplasia and compensatory hyperinflation of the left lung

**Figure 3:**
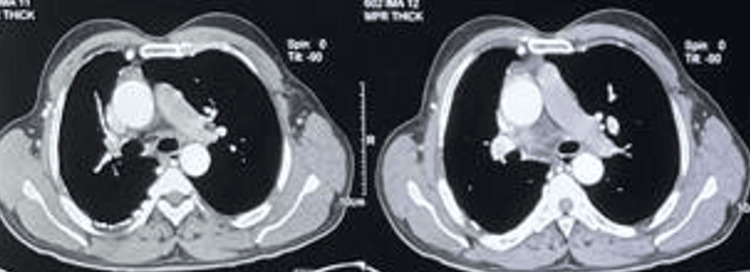
Mediastinal section thoracic CT scan: the absence of the right pulmonary artery and left-sided dominance of pulmonary blood flow

On clinical examination, he maintained an oxygen saturation of 98% on room air, with no abnormalities on auscultation. Other clinical examinations and laboratory analyses were normal. A chest X-ray (Figure 1) revealed volume loss in the right lung, hyperinflation on the left side, and poor visualization of the right hilar structures. A chest CT angiogram (Figures 2-3) was performed, which confirmed agenesis and lack of individualization of the right pulmonary artery, with ipsilateral pulmonary hypoplasia. This was associated with bronchial and non-bronchial bypass pathways (bronchocostal trunk, internal mammary arteries, right phrenic arteries, and a right subdiaphragmatic collateral), and bronchial thickening with bilateral micronodules of infectious appearance.

**Figure 4:**
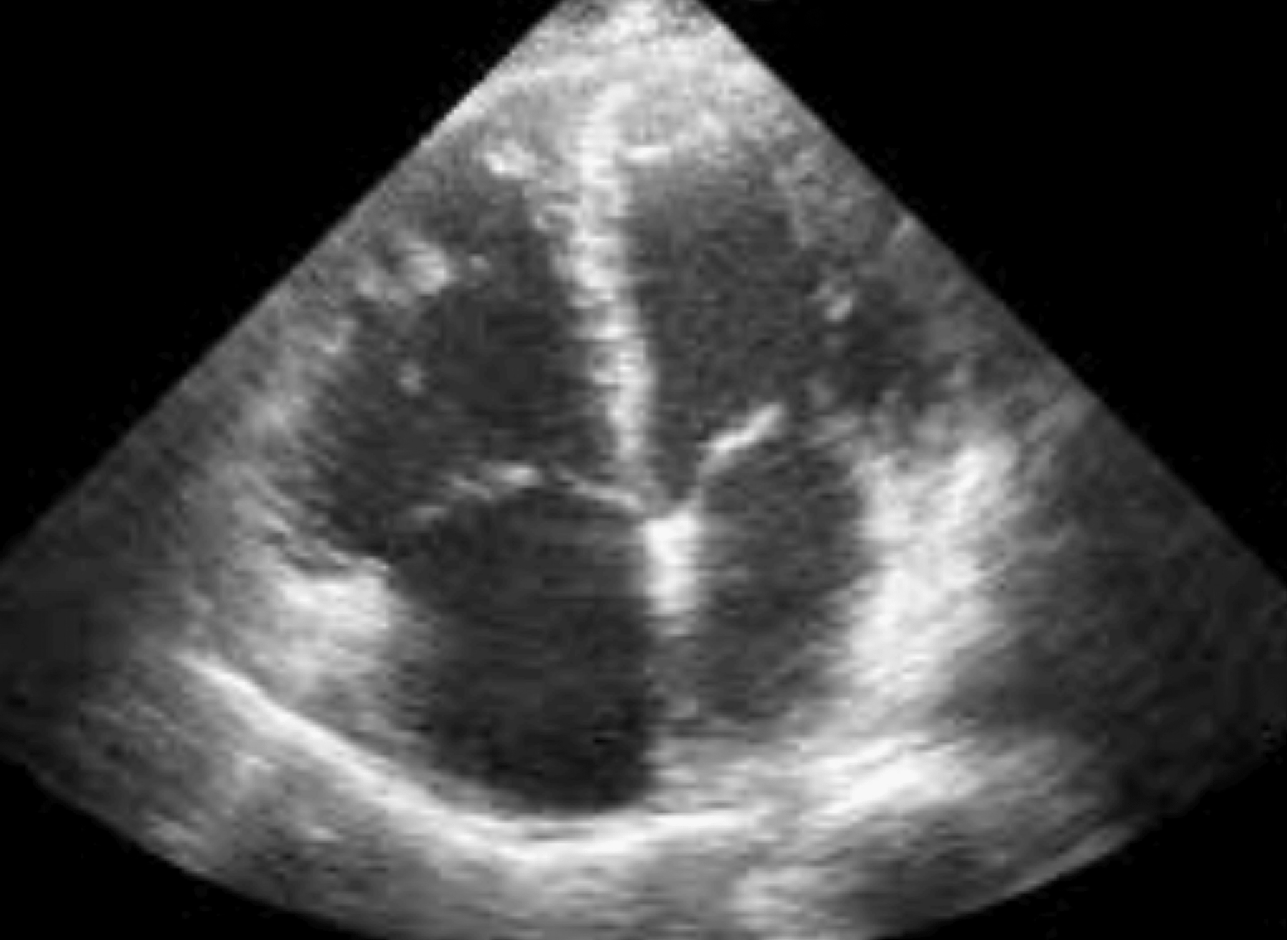
No signs of malformation were noted on transthoracic echocardiography

No signs of malformation were noted on transthoracic echocardiography (Figure 4); no pulmonary hypertension or patent ductus arteriosus was observed, no valvulopathies were found, and the cardiac chambers appeared normal. Plethysmography was at the limit of normal: forced expiratory volume in one second (FEV1) = 3.13 L (87% predicted), FEV1/forced vital capacity (FVC) = 89% predicted, and total lung capacity (TLC) = 4.94 L (80% predicted). After a multidisciplinary discussion, close clinical, functional, and radiological monitoring was opted for without immediate surgical intervention. The patient has been followed for one year with good progress.

## Discussion

UAPA results from premature obliteration of the proximal right terminal arch during embryogenesis. Persistence of the distal segment explains collateral formation and the brachiocephalic diverticulum. Traditional numbering of the pharyngeal arches (1-4 and 6) is debated. Some propose simplified numbering (1-5) or renaming the sixth arch as the "pulmonary arch" to clarify terminology [4].

Jariwala et al. cited rare cases of acquired UAPA, attributed to chronic subclinical infections, commonly tuberculosis, involving the lung hilum [5].

In a 2011 study involving 419 cases of congenital unilateral absence of the pulmonary artery, 182 were isolated, 60% of which involved the right pulmonary artery [3].

A more recent Chinese retrospective study (2014-2020), despite its modest sample size, analyzed 49 cases of UAPA diagnosed by echocardiography. It found a predominance on the left side (55%), with 92% of cases associated with other cardiovascular malformations. Only 8% of cases (4/49) were isolated UAPA, mostly on the right (75%) [6].

Regarding complications, pulmonary hypertension was present in 55% of cases. The initial diagnostic concordance rate by ultrasound was 55%, which improved to 83% after expert review. Thus, while echocardiography is useful, it heavily depends on the operator’s experience [3,5,6].

Clinical presentation in infants includes heart failure and pulmonary hypertension, whereas in adults, 13-30% are asymptomatic. Symptoms may include dyspnea (63.6%), hemoptysis (32%), or recurrent infections (27%). Some authors have also reported atypical chest pain [3,5,6].

UAPA is diagnosed based on clinical evaluation and cardiopulmonary imaging. Diagnosing isolated UAPA on chest X-ray is difficult; it should be considered in the presence of asymmetry of the hemifields and abnormalities of the vascular structures. The electrocardiogram (ECG) is usually normal in patients with asymptomatic, isolated UAPA.

Transthoracic echocardiography is a fundamental examination to exclude associated congenital heart diseases. Under good conditions, it can detect the absence of a unilateral pulmonary artery and plays an important role in the monitoring and early detection of pulmonary hypertension. Transesophageal echocardiography may also be necessary [3,5,6].

Pulmonary function tests (PFTs) may reveal obstructive and restrictive bronchopneumopathy with decreased diffusion capacity and reduced values in various parameters.

CT or magnetic resonance pulmonary angiography (MRPA) is considered the reference standard for diagnosis. MRPA is useful for diagnosing associated congenital cardiovascular anomalies, while CT angiography better detects underlying parenchymal pulmonary pathologies. Invasive (conventional) pulmonary angiography is reserved for patients undergoing interventional procedures [3,5-7].

Right heart catheterization allows for assessment of hemodynamic parameters, including reversibility of pulmonary arterial hypertension (PAH) [6].

Ventilation-perfusion (V/Q) lung scintigraphy in UAPA may show normal or mildly reduced ventilation, a complete unilateral perfusion defect, and V/Q mismatch.

Therapeutic recommendations are primarily based on case reports and small series, with no formal consensus guidelines. Authors propose categorizing patients into two main groups: 1) those with minimal or no symptoms, and no evidence of cardiopulmonary dysfunction, who require no specific treatment but should have regular follow-up, and 2) those with symptoms such as dyspnea on exertion, hemoptysis, or recurrent respiratory infections, who may require definitive or palliative treatment.

Early diagnosis through advanced imaging (CT/MRI) is crucial. Individualized management combining observation, interventional/surgical procedures (such as lobectomy or pneumonectomy for severe hemoptysis or recurrent infections, arterial reconstruction or conduit grafting if anatomy allows, or heart-lung transplantation in cases of right heart failure and severe pulmonary hypertension), and medical therapies (such as pulmonary vasodilators like sildenafil and bosentan, or oxygen therapy), can improve both quality of life and survival. Asymptomatic cases require long-term monitoring to detect emerging PAH. Data on long-term follow-up remain insufficient [3,5-7].

The overall mortality rate is estimated at 7%, primarily due to severe PAH, massive hemoptysis, or right heart failure [3,5-7].

## Conclusions

Unilateral isolated pulmonary artery agenesis in adults, though uncommon, should be considered in cases of unexplained exertional dyspnea, hemoptysis, or incidental thoracic imaging findings. Definitive diagnosis relies on CT or MR pulmonary angiography, supplemented by echocardiography to assess for pulmonary hypertension and rule out associated cardiac anomalies. Management must be tailored to symptom severity: regular surveillance for asymptomatic individuals; embolization and/or lung resection for recurrent hemoptysis; and pulmonary vasodilator therapy to mitigate PAH. Long-term multidisciplinary follow-up is essential to optimize patient outcomes and quality of life. The follow-up of these patients, especially in the long term, requires further study to support evidence-based recommendations.
